# A Disentangled Representation Based Brain Image Fusion *via* Group Lasso Penalty

**DOI:** 10.3389/fnins.2022.937861

**Published:** 2022-07-18

**Authors:** Anqi Wang, Xiaoqing Luo, Zhancheng Zhang, Xiao-Jun Wu

**Affiliations:** ^1^School of Artificial Intelligence and Computer Science, Jiangnan University, Wuxi, China; ^2^Advanced Technology and Research Institute, Jiangnan University, Wuxi, China; ^3^School of Electronic and Information Engineering, Suzhou University of Science and Technology, Suzhou, China

**Keywords:** deep learning, image fusion, medical brain image, disentangled representation, group lasso penalty

## Abstract

Complementary and redundant relationships inherently exist between multi-modal medical images captured from the same brain. Fusion processes conducted on intermingled representations can cause information distortion and the loss of discriminative modality information. To fully exploit the interdependency between source images for better feature representation and improve the fusion accuracy, we present the multi-modal brain medical image fusion method in a disentangled pipeline under the deep learning framework. A three-branch auto-encoder with two complementary branches and a redundant branch is designed to extract the exclusive modality features and common structure features from input images. Especially, to promote the disentanglement of complement and redundancy, a complementary group lasso penalty is proposed to constrain the extracted feature maps. Then, based on the disentangled representations, different fusion strategies are adopted for complementary features and redundant features, respectively. The experiments demonstrate the superior performance of the proposed fusion method in terms of structure preservation, visual quality, and running efficiency.

## 1. Introduction

Medical image fusion is an important branch of information fusion tasks. Typical types of medical images include Magnetic Resonance Imaging (MRI),Computed Tomography (CT), and Positron Emission Tomography (PET). MRI images are of high resolution and provide precise information about soft tissue, CT images provide dense structures like bones, and PET images assess the functions of organs and tissue. The objective of medical image fusion is to combine the complementary and redundant features from multi-modal medical images into one composite image with all the significant information, thus facilitating the process of clinical diagnosis. Image fusion methods can be generally divided into traditional ones and deep learning-based ones.

Traditional multi-scale transform (MST) based image fusion methods are popular in the community as the MST tools are able to simulate the human visual system to analyze the image, as well as to extract geometry structure and details of the image. Commonly adopted MST tools include discrete wavelet transform(DWT) (Ben et al., 2005), shift-invariant shearlet transform (Luo et al., 2016), and contourlet transform (Yang et al., 2010). Fused images with good quality can be obtained through the appropriate manual design of activity level measurements and fusion rules on the extracted features. However, to get better fusion performance, the manual design of fusion rules tends to become more and more complex, which results in higher computation costs.

Compared to the traditional methods, deep learning-based methods have been demonstrated with the great ability to automatically extract hierarchical and representative features of different abstraction levels. The typical deep learning model used for image fusion is Convolutional Neural Networks (CNN). Liu et al. (2017) applied CNN in image fusion, where the CNN predicts the importance of each pixel of source images. With the output decision map, source images are combined to get the fused image. Li et al. (2018) adopted the VGGNet pre-trained on the ImageNet dataset to extract the features from high frequency coefficients, which can effectively reflect the regions with abundant information. While these methods partially depend on the CNN and extra manual processes are required. To realize the end-to-end image fusion process, some unsupervised CNN-based methods and Generative adversarial Network (GAN) based methods are proposed subsequently (Huang et al., 2020; Ma et al., 2020; Xu and Ma, 2021; Guo et al., 2022; Xu et al., 2022). As an example for each category, Xu and Ma (2021) adopted both subjectively defined features and deep features to measure the activity level of input images, then adaptive weights can be assigned to loss functions to adjust the similarity between the fused image and each source image; Ma et al. (2020) proposed the DDcGAN which establishes the adversarial relationships between a generator and two discriminators to introduce abundant information from the source images of both modalities. Another popular pipeline for image fusion is to fuse the deep features extracted from an auto-encoder which has great feature extraction and image reconstruction abilities (Li and Wu, 2018; Li et al., 2020; Jian et al., 2021). Even though state-of-the-art performance has been achieved, the above methods leverage the same feature representation for different modalities to design the fusion rule or directly fuse the multi-modal features in an intermingled way, thus they cannot fully exploit the prior knowledge of complementary and redundant contained in multi-modal images. Redundant information is the common type of features such as structure and shape, while complementary information represents the most unique characteristics belonging to one specific modality, which is hierarchical and hard to represent by hand-crafted features. Thereby, fusion operations conducted on intermingled representations can cause the degradation of discriminative features and the introduction of distorted information.

The criteria for learning good representations discussed in Bengio et al. (2013) show that one of the important points is to disentangle the variable features for the explanatory factors. If exclusive representations can be obtained for multi-modal images to separate the complementary and redundant features, then the more interpretable representations can improve the accuracy of the fusion decision. Recently, some work has researched the disentanglement representations for image fusion. Xu et al. (2021) disentangled the features of infrared and visible images into attribute and scene modality, for each the weighted average fusion rule is adopted. Luo et al. (2021) believed that all kinds of paired source images share the private and common features, and proposed a general framework for image fusion that takes advantage of contrastive learning for better disentanglement. In the above two studies, the attribute and private features are exactly the complementary ones, while the scene and common features are the redundant ones. Both of them have alleviated the pressure of designing appropriate fusion strategies and achieved good fusion performance. However, there still exist some problems: (1) In Xu et al. (2021), the attribute modality is compressed into a vector, resulting in the loss of spatial information and lack of interpretability. Thereby, the weighted-average fusion rule on the attribute representation leads to blur results and information distortion. (2) Xu et al. (2021) force the infrared and visible attribute distribution close to a prior Gaussian distribution, while Luo et al. (2021) minimize the cosine similarity among private and common representations. Both of them lack the consideration of the importance of features in the local position of both source images, thus weakening the ability of disentangled representations to present the most meaningful information.

In order to achieve a more robust and controllable fusion decision, we aim to incorporate the explicit constraints on the deep feature maps extracted by the encoder. In the field of machine learning, feature selection is an important stage to reduce the data dimension and determine the relevant features for a specific learning task. Recently, sparsity-inducing regularization techniques are widely adopted in feature selection methods to filter out the irrelevant features from multiple heterogeneous feature descriptors (Zhao et al., 2015). Li et al. (2019) proposed an adaptive sparse group lasso penalty on the clustered genes to select the biologically significant genes. To control the attention response and restrain the noisy information, Wang and Guo (2021) applied sparse regularization on the computed attention maps. Considering the redundancy may exist among features, Wang et al. (2021) proposed using Group lasso to prevent the selection of redundant features which may have high correlations with other features. Inspired by these studies, we think the learning process of complementary and redundant representations can also be realized through the regularization techniques on the extracted feature maps to filter out the complementary features from the redundant ones.

Based on the above considerations, we propose a disentangled representation based brain image fusion method *via* group lasso penalty. A three-branch auto-encoder with two complementary branches and one redundant branch is designed to deal with the unique modality characteristics and common structure information inherent in the multi-modal source images. In the training stage, the auto-encoder should be able to reconstruct both source images conditioned on the extracted complementary features and redundant features. For effective disentangled representation learning, a complementary group lasso penalty is proposed to restrain the redundant information in the complementary features, promoting the complementary encoders to learn the most discriminative information. In the fusion stage, different fusion strategies are adopted for complementary and redundant features respectively. Then, the fused image can be obtained by reconstructing from the fused features. To sum up, the contributions of the proposed method are as follows:

- A disentangled representation based brain image fusion method is proposed to fully exploit the redundancy and complement prior relationships among multi-modal source images.
- A complementary group lasso penalty is designed to promote the disentanglement ability and ensure the complementary feature maps of significant modality information.
- Comparison experiments conducted on MRI-CT and MRI-PET fusion tasks with state-of-the-art deep learning-based methods demonstrate the superior fusion performance of the proposed method quantitatively and qualitatively.

The remaining part of the article is organized as follows. Section 2 briefly introduces the definition of group lasso penalty. Section 3 describes the proposed method in detail. The experiment results are shown in Section 4. The conclusion and an outlook of future study is presented in Section 5. The implementation code of the proposed model will be available on our project page.

## 2. Group Lasso Penalty

In 2006, Yuan (2006) proposed the group lasso penalty in a linear model, which aims to select the grouped explanatory variables for the accurate prediction of a regression problem. Given a response variable *y*∈*R*^*N*^, a feature matrix *X*∈*R*^*N* × *P*^, and a coefficient vector β∈*R*^*P*^, where *P* is the number of feature variables and *N* is the number of observation values, the objective of the group lasso estimation model is defined as follows:


(1) $$\underset{\beta \in R^{p}}{\arg\min}\frac{1}{2}\|y-X\beta {\|}_{2}^{2}+\text{λ}\sum_{l=1}^{L}\sqrt{p_{l}}\|\beta_{l}{\|}_{2}.$$

Here, the first term is the loss function and the second term is the group lasso penalty. *P* feature variables are further divided into *L* sub groups, each group contains *p*_*l*_ variables, β_*l*_ is the coefficient sub vector corresponding to the *l*^*th*^ group (*l* = 1, 2, ..., *L*), λ≥0 is a tuning parameter, ∥ · ∥_2_ is the L2 norm. Group lasso penalty is able to exploit the group structure of variables and promote the selection of the most relevant feature variables, thus simplifying a model, avoiding overfitting, and enhancing the interpretability of a model.According to the context and requirements of a specific task, the loss function, grouping situation, and λ can be adjusted. Inspired by the effectiveness of the group lasso penalty in selecting significant features, we consider the feature vectors of different pixel positions in a feature map can be regarded as a feature waiting to be penalized, and a task like an image reconstruction can be regarded as the loss function in Equation 1. The difference is that the penalty in Equation 1 is imposed on the coefficients, while in this paper, the penalty is directly imposed on the extracted feature maps to filter out the redundant features from the complementary ones, thus promoting the accuracy of disentangled representations.

## 3. Frames and Methods

In this section, a detailed description of the disentangled representation based image fusion framework is given first. Then, the design of the loss functions and the adopted fusion strategies are described, respectively.

### 3.1. Overall Framework

**Figure 1:**
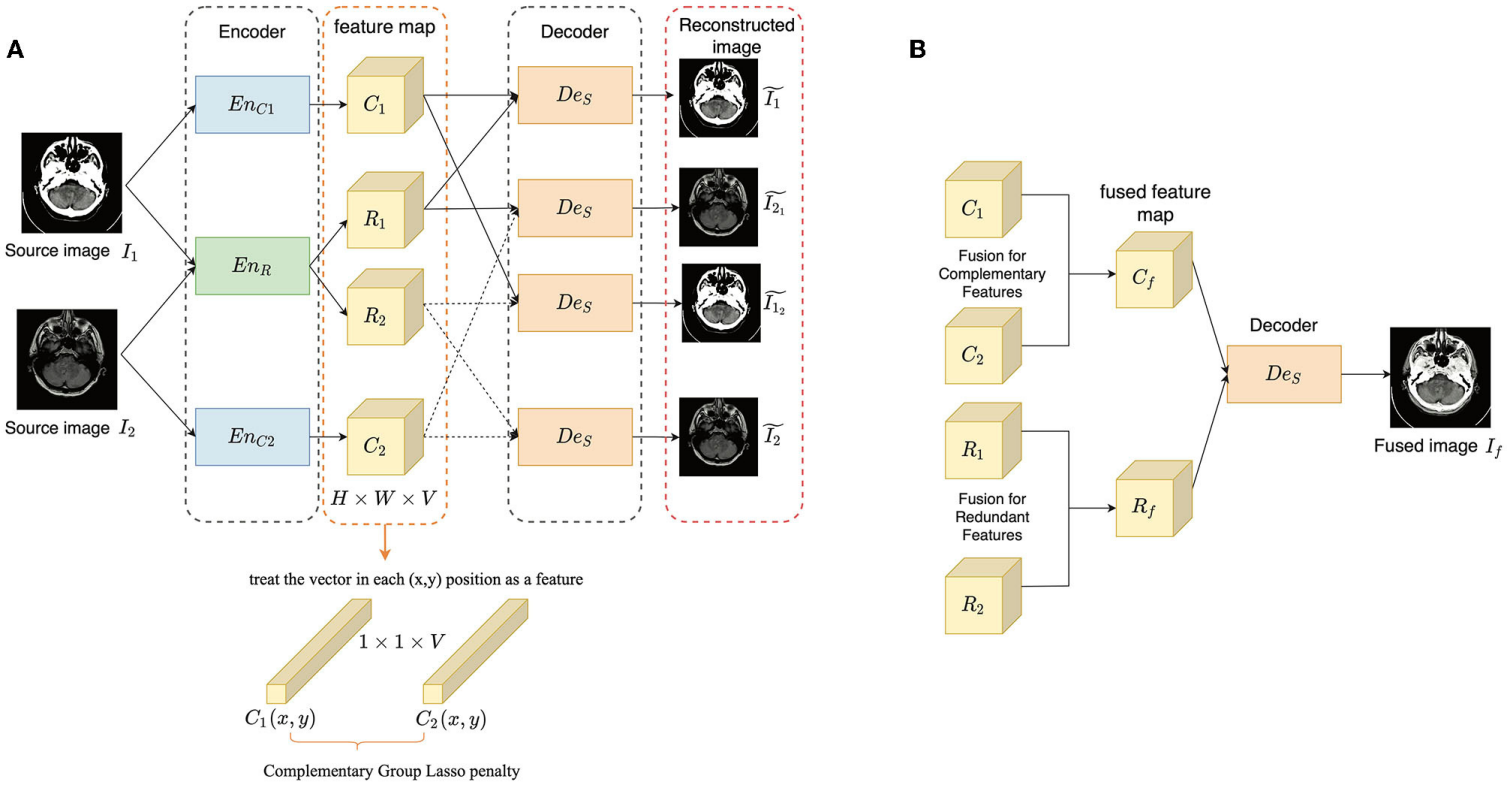
The overview of the proposed method: **(A)** the training stage; **(B)** the fusion stage. The encoder-decoder architecture contains two complementary encoders *En*_*C*1_ and *En*_*C*2_, one redundant encoder *En*_*R*_ and one shared decoder *De*_*S*_. The extracted complementary and redundant features of the two source images are denoted as C_*_ and R_*_(*∈1, 2), and each of them is of size *H* × *W* × *V*.

The aim of the proposed method is to separate the complementary features from the redundant features for each modality, thus improving the interpretability of feature representation and the fusion accuracy. The overall framework of the proposed method is illustrated in Figure 1, which includes a training stage (Figure 1A) and a fusion stage (Figure 1B). The training stage is to train an auto-encoder to learn disentangled representation and image reconstruction ability, while the fusion stage is to get the fused image through fusing the disentangled representations. We denote that the input source images from two different modalities as *I*_1_ and *I*_2_, respectively. Since the complementary features contain the discriminative modality information and the redundant features contain the common structure information, two complementary encoders *En*_*C*1_ and *En*_*C*2_ is used to extract the unique information, respectively, and one shared redundant encoder *En*_*R*_ is designed to map the structure information into a common space.

In the training stage, *I*_1_ and *I*_2_ are encoded by the three encoders to get complementary and redundant features, respectively as follows:


(2) $$\begin{array}{r} \left\{C_{*},R_{*}\right\}=\left\{En_{C*}\left(I_{*}\right),En_{R}\left(I_{*}\right)\right\},*\in \left\{1,2\right\}, \end{array}$$

where C_*_ and R_*_ are the complementary and redundant features of I_*_. Then, the input images should be able to be reconstructed from the combined features as follows:


(3) $$\begin{array}{r} \tilde{I_{*}}=De_{S}\left(C_{*}+R_{*}\right),*\in \left\{1,2\right\}, \end{array}$$

where $\tilde{I_{*}}$ is the reconstructed version of I_*_. Besides, as the complementary features are expected to represent the most unique modality information and determine the appearance of an image, the output image should be as similar as possible to the input source image which provides the complementary features. The process is described as follows:


(4) $$\begin{array}{r} \begin{array}{c} \tilde{I_{1_{2}}}=De_{S}\left(C_{1}+R_{2}\right),\\ \tilde{I_{2_{1}}}=De_{S}\left(C_{2}+R_{1}\right), \end{array} \end{array}$$

where $\tilde{I_{1_{2}}}$ is the reconstructed image *I*_1_ conditioned on *C*_1_ and *R*_2_, while $\tilde{I_{2_{1}}}$ has a similar definition. To achieve good image reconstruction ability, Mean Square Error (MSE) and Structural Similarity (SSIM) (Wang et al., 2004) are adopted as the image reconstruction loss. Only using the shared-weight strategy in *En*_*R*_ cannot guarantee the disentanglement, we adopted two kinds of constraints to improve the disentangled representation learning: a complementary group lasso penalty term and a redundant consistency constraint term, which are introduced in Section 3.2. The former is adopted to restrain the growth of redundant information in the extracted complementary feature maps, while the latter is designed based on the assumption that the multi-modal images captured in the same scene should share as much structure information as possible.

In the fusion stage, the complementary and redundant features are extracted from source images firstly as in the training stage, while before combining them, different fusion strategies (Section 3.3) are defined for them. After obtaining the fused complementary and redundant feature (*C*_*f*_ and *R*_*f*_), they are added together and input to *De*_*S*_ to get the final fused image *I*_*f*_ as follow:


(5) $$\begin{array}{r} \tilde{I_{f}}=De_{S}\left(C_{f}+R_{f}\right). \end{array}$$

The input images are assumed as gray scale images. If the input is an *RGB* image, it is first converted into *YCbCr* color space, and the *Y*(luminance) component is used for fusion. After getting the gray scale fused image, it is combined with *Cb* and *Cr*(chrominance) components and inversely converted into the *RGB* fused image.

As for the network architecture, in each encoder, there are three 3 × 3 convolutional blocks with *ReLU* activation, except for the first one, each followed by a Batch Normalization layer. The weights of the first three layers in VGG-19 (Simonyan and Zisserman, 2015) are used to initialize the complementary and redundant encoders, as VGG-19 is a well-trained feature extractor that can relieve the training pressure. The architecture of the decoder is symmetric as the encoder, while in the output layer, Sigmoid is adopted as the activation function to constrain the value between [0,1]. Detailed information about the network is shown in Table 1.

**Table 1:** The architecture of encoder and decoder.

	**Layer**	**Size**	**Stride**	**Channel**	**Channel**	**Activation**	**Normalization**
				**(input)**	**(output)**		
Encoder	Conv1	3 x 3	1	1	64	ReLU	/
	Conv2	3 x 3	1	64	64	ReLU	Batch
	Conv3	3 x 3	1	64	128	ReLU	Batch
Decoder	Conv1	3 x 3	1	128	64	ReLU	Batch
	Conv2	3 x 3	1	64	64	ReLU	Batch
	Conv3	3 x 3	1	64	1	Sigmoid	/

*Conv means the convolutional block with activation and normalization layer*.

### 3.2. Loss Function

**1) Complementary group lasso penalty term:** The extracted feature maps are considered with the size of *H* × *W* × *V*, where *H*, *W*, and *V* correspond to the height, width, and channel dimensions, respectively. Each 1 × 1 × *V* vector in position (*x, y*) is treated as a feature waiting to be penalized. We denote the features of *I*_1_ and *I*_2_ extracted by the complementary encoder in position (*x, y*) as *C*_1_(*x, y*) and *C*_2_(*x, y*). To determine the type of a feature, the similarity between *C*_1_(*x, y*) and *C*_2_(*x, y*) is computed by cosine similarity as follows:


(6) $$\begin{array}{r} \begin{array}{c} r\left(x,y\right)=\frac{C_{1}\left(x,y\right)\cdot C_{2}\left(x,y\right)}{∥C_{1}\left(x,y\right){∥}_{2}∥C_{2}\left(x,y\right){∥}_{2}}, \end{array} \end{array}$$

The high similarity means the information is redundant, on the contrary, complementary. The importance ϕ_*_ of a feature is measured based on the *L*1 norm and average operator in a local block around C_*_(*x, y*) as follow:


(7) $$\begin{array}{r} \begin{array}{c} \phi_{*}\left(x,y\right)=\frac{\sum_{i=-r}^{r}\sum_{j=-r}^{r}{Ĉ}_{*}\left(x+i,y+j\right)}{{\left(2r+1\right)}^{2}}, \end{array} \end{array}$$

where Ĉ_*_(*x, y*) is the *L*1 norm of C_*_(*x, y*) computed as follows:


(8) $$\begin{array}{r} \begin{array}{c} {Ĉ}_{*}\left(x,y\right)=∥C_{*}\left(x,y\right){∥}_{1}. \end{array} \end{array}$$

Then, a complementary Group lasso penalty *L*_*c*_ is proposed to restrain the redundancy and promote complement in *C*_1_ and *C*_2_:


(9) $$\begin{array}{r} \begin{array}{c} L_{c}=\overset{W\times H}{\underset{i=1}{\sum }}\left(\omega_{1}∥C_{1}\left(x,y\right){∥}_{2}+\omega_{2}∥C_{2}\left(x,y\right){∥}_{2}\right), \end{array} \end{array}$$

where ω_1_ and ω_2_ are defined in the form of a Sigmoid function as follows:


(10) $$\begin{array}{l} \omega_{1}=\frac{1}{1+exp(k(\phi_{2}(x,y)-\phi_{1}(x,y)))},\\ \omega_{2}=1-\omega_{1}. \end{array}$$

In Equation (10), *k* is the parameter that controls the shape of the function and is defined based on the similarity:


(11) $$\begin{array}{r} \begin{array}{c} k=\frac{1}{r^{2}\left(x,y\right)}. \end{array} \end{array}$$

The smaller the similarity between *C*_1_(*x, y*) and *C*_2_(*x, y*) is, the larger the *k* is. Then, the shape of the sigmoid function becomes steeper.

**Figure 2:**
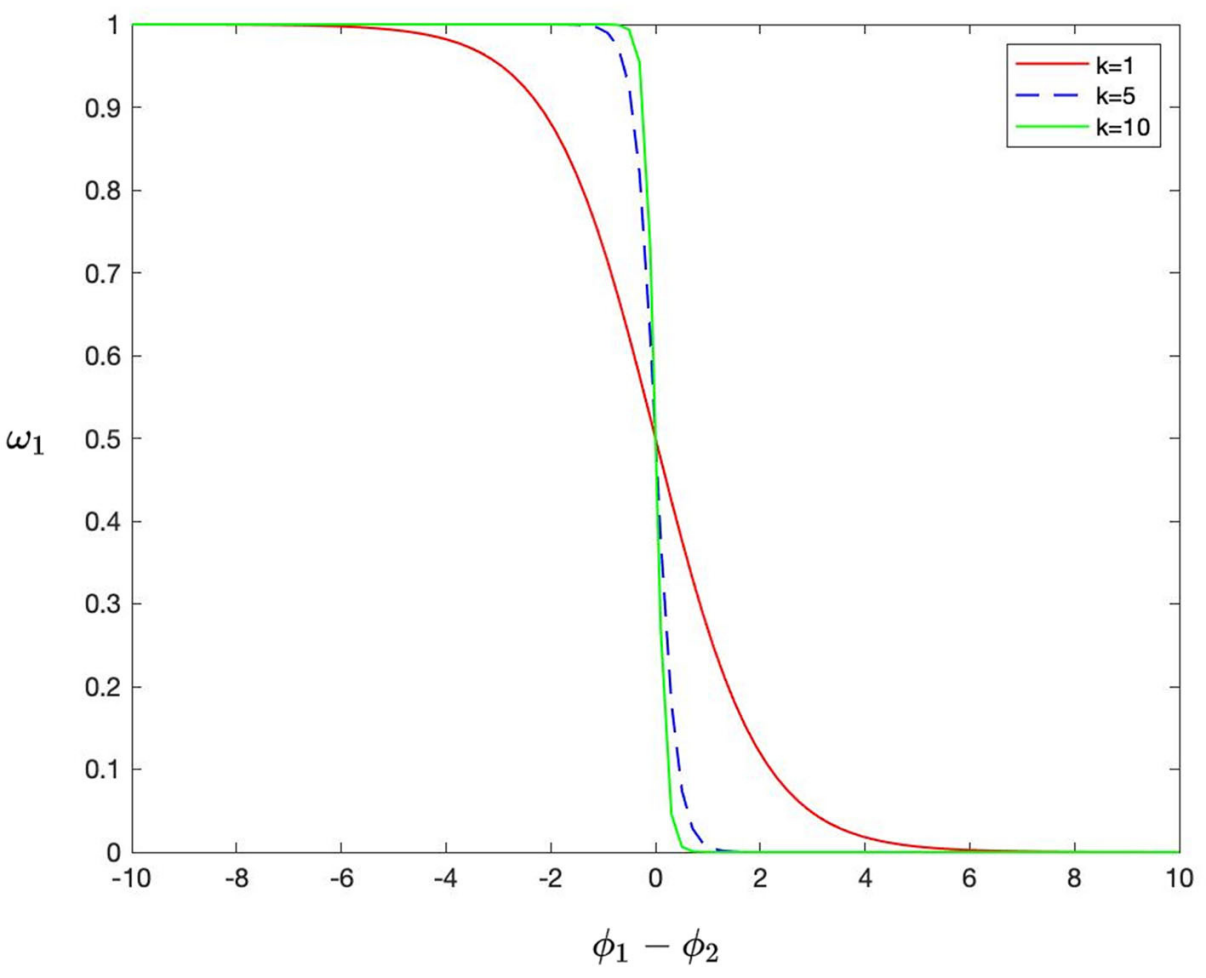
The shape of the function of ω_1_.

Figure 2 shows the function of ω_1_ in Equation 10. The smaller the similarity is, the closer the weight assignment is to choose-max, on the contrary, close to average-weighting. Then, the weight value is further determined by the ϕ_*_(*x, y*). When Equation 9 is going to be minimized in an iteration if ϕ_1_(*x, y*) is much larger than ϕ_2_(*x, y*), which means *C*_1_(*x, y*) is much more important than *C*_2_(*x, y*). At this time, ϕ_1_(*x, y*)−ϕ_2_(*x, y*) is a positive value, and ω_1_ tends to become zero. Then, less penalty is imposed on *C*_1_(*x, y*), while *C*_2_(*x, y*) is greatly penalized and filtered out from the complementary feature maps. On the contrary, *C*_1_(*x, y*) is greatly penalized. If *C*_1_(*x, y*) is similar to *C*_2_(*x, y*), it means they share a lot of redundant information, and ϕ_1_(*x, y*)−ϕ_2_(*x, y*) becomes close to zero. Thereby both of them are equally penalized and gradually pushed into *R*_1_(*x, y*) and *R*_2_(*x, y*). Finally, the complementary feature maps should contain the most significant modality characteristics.

**2) Redundant consistency constraint term:** As the multi-modal medical images are captured from the same brain, they must contain redundant information like structure and shape. It is expected that both *R*_1_ and *R*_2_ maintain a similar information. However, the multi-modal medical images provide an unequal amount of information, and they show their own biases toward some specific parts of the brain. Moreover, a shared *En*_*R*_ is adopted to extract the redundant feature, thus *R*_1_ and *R*_2_ cannot be the same. Compared to constraining the similarity of the extracted features, the redundant consistency constraint term *L*_*r*_ is conducted on the reconstructed results of *R*_1_ and *R*_2_ as follows:


(12) $$\begin{array}{r} \begin{array}{c} L_{r}=∥De_{S}\left(R_{1}\right)-De_{S}\left(R_{2}\right){∥}_{1}, \end{array} \end{array}$$

**3) Image reconstruction loss:** The image reconstruction loss is to enforce the output images to have high reconstructed precision with the input images, thus ensuring that the auto-encoder has both good feature extraction and image reconstruction ability. The image reconstruction loss *L*_*rec*_ is defined based on pixel loss *L*_*MSE*_ and SSIM (Wang et al., 2004) *L*_*SSIM*_ is as follows:


(13) $$\begin{array}{r} \begin{array}{cc} L_{MSE} & =∥I_{1}-\tilde{I_{1}}{∥}_{2}+∥I_{1}-\tilde{I_{1_{2}}}{∥}_{2}+∥I_{2}-\tilde{I_{2}}{∥}_{2}+∥I_{2}-\tilde{I_{2_{1}}}{∥}_{2},\\ L_{SSIM} & =\left(1-SSIM\left(I_{1},\tilde{I_{1}}\right)\right)+\left(1-SSIM\left(I_{1},\tilde{I_{1_{2}}}\right)\right)\\ \;+\left(1-SSIM\left(I_{2},\tilde{I_{2}}\right)\right)+\left(1-SSIM\left(I_{2},\tilde{I_{2_{1}}}\right)\right),\\ L_{rec} & =\lambda_{SSIM}L_{SSIM}+L_{MSE}, \end{array} \end{array}$$

where λ_*SSIM*_ is the parameter to balance the pixel loss and SSIM loss.

Thus, the overall loss is defined as follows:


(14) $$\begin{array}{r} \begin{array}{c} L=L_{rec}+\lambda_{r}L_{r}+\lambda_{c}L_{c}, \end{array} \end{array}$$

where λ_*r*_ and λ_*c*_ are the parameters to control the tradeoff of *L*_*r*_ and *L*_*c*_.

### 3.3. Fusion Strategy

The complementary features are exclusive for each modality, here, three kinds of fusion strategies are considered, including the addition strategy, max-selection strategy, and *L*1-norm strategy. Their impact on the results is compared in Section 4. The addition strategy is formulated as follows:


(15) $$\begin{array}{r} \begin{array}{c} C_{f}\left(x,y\right)=C_{1}\left(x,y\right)+C_{2}\left(x,y\right), \end{array} \end{array}$$

The max-selection strategy preserves the features of higher magnitude and is formulated as follows:


(16) $$C_{f}(x,y)=\{\begin{array}{ll} C_{1}(x,y)\;, & C_{1}(x,y)\geq C_{2}(x,y),\\ C_{2}(x,y)\;,\; & C_{2}(x,y)<C_{1}(x,y). \end{array}$$

The *L*1-norm strategy is designed based on the importance of each pixel position to adjust the information preservation degree of each source image. The L1-norm of complementary feature maps is computed as Equation 8 and is treated as the activity level measurement A_*_(*x, y*), *∈{1, 2}, then, the *L*1-norm strategy is formulated as follows:


(17) $$\begin{array}{r} \begin{array}{cc} C_{f}\left(x,y\right) & =\mu_{1}\times C_{1}\left(x,y\right)+\mu_{2}\times C_{2}\left(x,y\right), \end{array} \end{array}$$

where


(18) $$\begin{array}{l} \mu_{1}=\frac{A_{1}(x,y)}{A_{1}(x,y)+A_{2}(x,y)},\\ \mu_{2}=1-\mu_{2}. \end{array}$$

The redundant information is mapped to the same space, thereby, a simple average strategy is adopted as follows:


(19) $$\begin{array}{r} \begin{array}{c} R_{f}=\frac{R_{1}\left(x,y\right)+R_{2}\left(x,y\right)}{2}. \end{array} \end{array}$$

The final fused image is reconstructed by decoding the added *C*_*f*_ and *R*_*f*_.

## 4. Experiments and Analyses

In this section, we compare the proposed method with several typical deep learning-based image fusion methods on MRI-CT and MRI-PET image fusion tasks. First, the ablation study is conducted on the proposed complementary group lasso penalty term to verify its effectiveness. Then, the comparative study is conducted qualitatively and quantitatively. Finally, the time cost comparison of different methods is also conducted.

### 4.1. Experimental Settings

The training and testing dataset is built on the Harvard medical dataset (Summers, 2003), providing a brain image with a size 256 × 256. The slices with effective information are selected and there are a total of 180 pairs of MRI-CT images and 260 pairs of MRI-PET images. Considering that the number of the image is limited, when in the training phase, 10-fold verification experiments are performed and all the input images are randomly cropped into image patches of size 120 × 120, as well as randomly flipped and rotated. The setting of parameters are as follows: the batchsize is 8, the learning rate is 1e-4, and the size of a local block to measure the importance of a feature is 3, thus *r* is defined as 1. The other parameters like λ_*SSIM*_, λ_*r*_, and λ_*c*_ are set as 1,000, 10, and 10. The proposed method was implemented in Pytorch, and all experiments are conducted on a platform with Intel Core i7-6850K CPU and GeForce GTX 1080Ti GPU.

In the testing phase, the proposed method is compared with 6 deep learning-based methods, including CNN based methods EMFusion (Xu and Ma, 2021), U2Fusion (Xu et al., 2022), GAN based method DDcGAN (Ma et al., 2020), auto-encoder based method IFSR (Luo et al., 2021), DRF (Xu et al., 2021), and SEDR (Jian et al., 2021). All the code of the comparison methods are publicly available and the parameter settings are set according to the reference paper. Besides, the proposed method takes three different fusion strategies for the complementary features and they are also compared, which are denoted as proposed-add, proposed-max, and proposed-l1, respectively. For the proposed method, the average results of the quantitative evaluations and their corresponding variances of the 10 groups of the multi-fold verification experiments are presented in the table. The other comparison methods are also tested on the 10 groups respectively and the average values and variances of the 10 groups are computed.

### 4.2. Objective Metrics

Eleven objective metrics are adopted to conduct a comprehensive evaluation, including standard deviation (*SD*), spatial frequency (*SF*) (Ma et al., 2019), normalized mutual information (*Q*_*MI*_) (Hossny et al., 2008), nonlinear correlation information entropy (*Q*_*NCIE*_) (Qiang et al., 2005), gradient-based fusion performance (*Q*_*G*_) (Xydeas and Pv, 2000), a multiscale scheme based metric (*Q*_*M*_) (Wang and Liu, 2008), Piella's Metric (*Q*_*S*_) (Piella and Heijmans, 2003), multi-scale structural similarity (*MSSSIM*) (Ma et al., 2015),the sum of the correlations of differences (*SCD*) (Aslantas and Bendes, 2015), Chen-Blum Metric (*Q*_*CB*_) (Chen and Blum, 2009), and visual information fidelity based method (*VIFF*) (Han et al., 2013). Among them, *SD* reveals the distribution of gray levels and reflects the contrast of an image. *SF* measures the vertical and horizontal gradients, reflecting the changes in texture. *Q*_*MI*_ measures the amount of information transferred from source images to the fused images. *Q*_*NCIE*_ reveals the nonlinear correlation between source images and fused images. *Q*_*G*_ measures the amount of edge information transferred from source images to the fused images, while *Q*_*M*_ measures the amount of multi-scale edges. Both *Q*_*S*_ and *MSSSIM* reflect the structural similarity between source images and fused image, as well as quantifying the perceived distortion, while the former is edge-dependent and the latter is conducted based on multi-scale decomposition. *SCD* reveals how the complementary information is obtained by the fused image from source images. *Q*_*CB*_ and *VIFF* are human perception inspired metrics. *Q*_*CB*_ measures the similarity between source images and fused images based on the characteristics of a human visual system such as contrast and masking phenomenon, while *VIFF* measures the effective visual information contained in the fused image based on the natural scene statistics theory. A larger value of all the mentioned metrics corresponds to a good fusion performance.

### 4.3. Ablation Study

**Figure 3:**
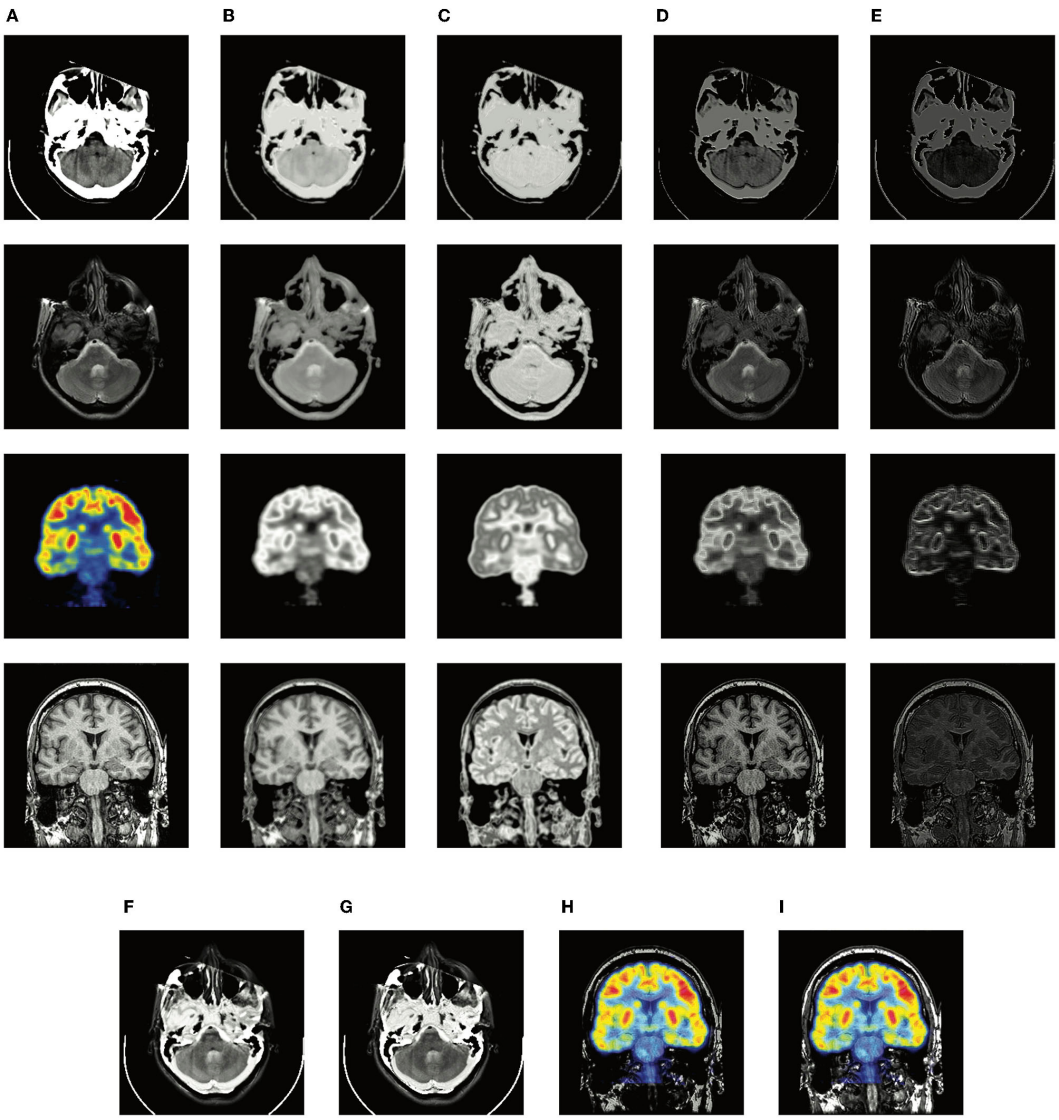
The illustration of visualized feature maps and fused results without and with complementary group lasso penalty term *L*_*c*_. **(A)** source images; **(B)** the redundant features of the proposed method without *L*_*c*_; **(C)** the redundant features of the proposed method; **(D)** the complementary features of the proposed method without *L*_*c*_; **(E)** the complementary features of the proposed method; **(F,H)** the fused results of the proposed-add without *L*_*c*_; **(G,I)** the fused results of the proposed-add.

In this section, we verify the effectiveness of the complementary group lasso penalty term *L*_*c*_. The proposed method trained without *L*_*c*_ is denoted as the proposed method without *L*_*c*_, and the fusion evaluation is conducted based on the addition strategy. In Figure 3, the extracted feature maps of one MRI-CT sample and one MRI-PET sample is presented. It can be seen that the proposed method without *L*_*c*_ provides the redundant and complementary features (Figures 3B,C) quite similar to the source images, but with different pixel intensity, which means a relatively weak disentanglement ability. On the contrary, *L*_*c*_ is able to promote the disentanglement and extract the complementary features with sharper details (Figure 3E). From the fused results in Figures 3F–I, the edge and texture of (Figures 3F,H) are a bit blur, and (Figure 3H) loses a lot of MRI information. We also present the corresponding quantitative evaluation in Tables 2, 3. *L*_*c*_ is able to improve the performance on almost all the metrics. In the MRI-PET task, proposed without *L*_*c*_ achieves the best *Q*_*CB*_, which reveals that the fused results should have good visual contrast, while the results of the rest metrics show that there is much loss of details and structural information. The ablation study demonstrates the function of *L*_*c*_ to better exploit the complementary and redundant relationships among multi-modal images.

**Table 2:** The quantitative evaluation of the proposed method (addition strategy) without and with *L*_*c*_ on the MRI-CT dataset.

**Methods**	**Objective metrics**										
	***SD***	***SF***	***Q*_*MI*_**	***Q*_*NCIE*_**	***Q*_*G*_**	***Q*_*M*_**	***Q*_*S*_**	***MSSSIM***	***SCD***	***Q*_*CB*_**	***VIFF***
proposed-add	80.38 ± 2.12	25.41 ± 1.30	0.78 ± 0.02	0.81 ± 0.00	0.68 ± 0.02	0.14 ± 0.01	0.65 ± 0.23	0.91 ± 0.01	1.12 ± 0.09	0.54 ± 0.19	0.43 ± 0.01
without Lc											
proposed-add	84.92 ± 2.64	27.23 ± 0.27	0.80 ± 0.01	0.81 ± 0.00	0.72 ± 0.01	0.16 ± 0.02	0.82 ± 0.01	0.91 ± 0.01	1.37 ± 0.10	0.66 ± 0.01	0.45 ± 0.03

*The results of the proposed method are shown as average±variance of 10-fold verification experiments*.

**Table 3:** The quantitative evaluation of the proposed method (addition strategy) without and with *L*_*c*_ on the MRI-PET dataset.

**Methods**	**Objective metrics**										
	***SD***	***SF***	***Q*_*MI*_**	***Q*_*NCIE*_**	***Q*_*G*_**	***Q*_*M*_**	***Q*_*S*_**	***MSSSIM***	***SCD***	***Q*_*CB*_**	***VIFF***
proposed-add	80.20 ± 1.75	26.19 ± 1.78	0.65 ± 0.01	0.81 ± 0.00	0.62 ± 0.02	0.17 ± 0.02	0.76 ± 0.02	0.91 ± 0.01	1.36 ± 0.05	0.58 ± 0.01	0.52 ± 0.01
without Lc											
proposed-add	89.28 ± 1.21	34.41 ± 0.55	0.76 ± 0.01	0.81 ± 0.00	0.77 ± 0.00	0.51 ± 0.08	0.80 ± 0.01	0.94 ± 0.00	1.65 ± 0.03	0.50 ± 0.01	0.59 ± 0.00

*The results of the proposed method are shown as average±variance of 10-fold verification experiments*.

### 4.4. Qualitative Evaluation

**Figure 4:**
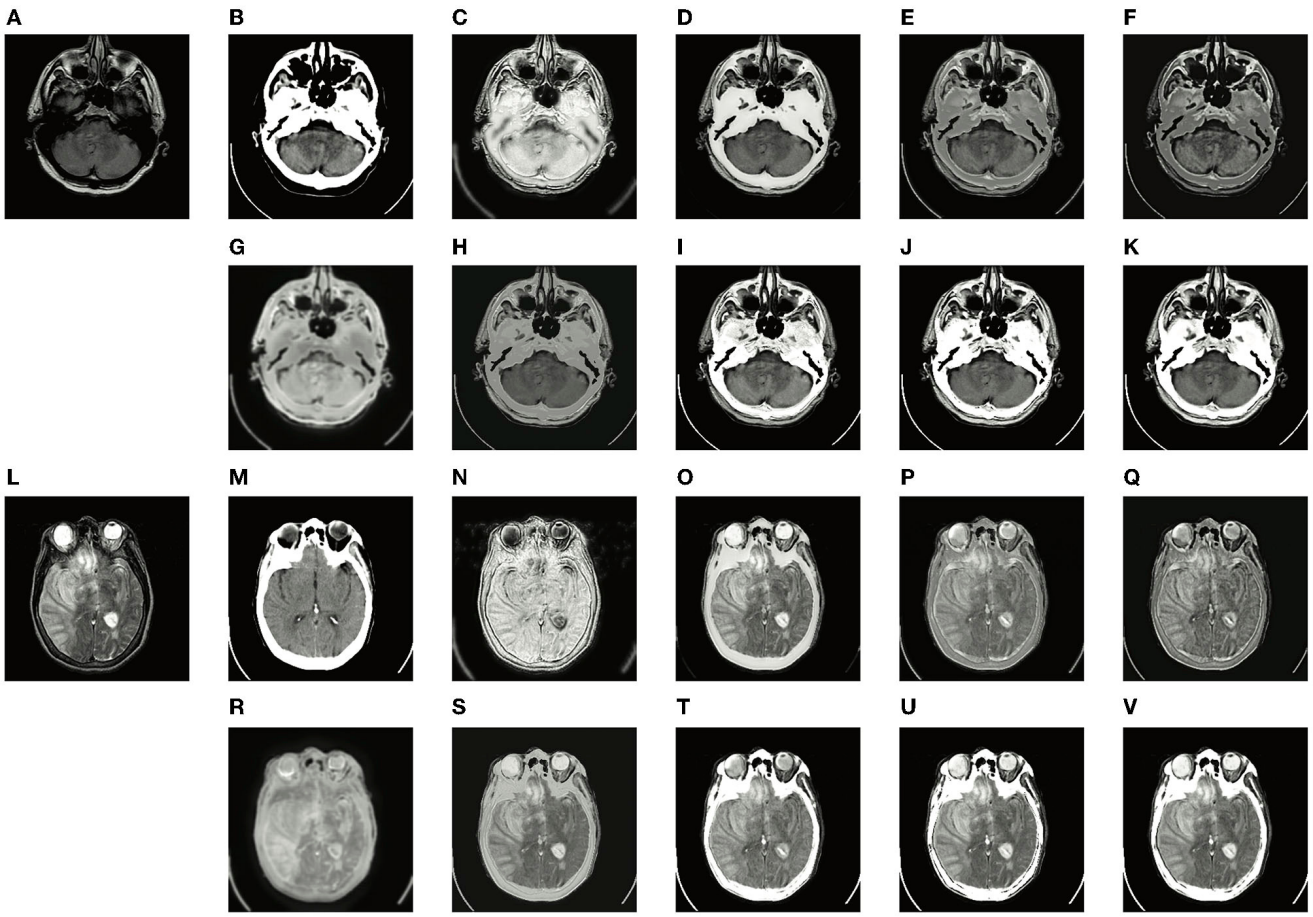
Experiments results of the proposed method with six deep learning-based methods on two typical MRI and CT image pairs. **(A,L)** MRI images; **(B,M)** CT images; **(C,N)** fused results of DDcGAN; **(D,O)** fused results of EMFusion; **(E,P)** fused results of IFSR; **(F,Q)** fused results of U2Fusion; **(G,R)** fused results of DRF; **(H,S)** fused results of SEDR; **(I,T)** fused results of proposed-add; **(J,U)** fused results of proposed-max; and **(K,V)** fused results of proposed-l1.

**Figure 5:**
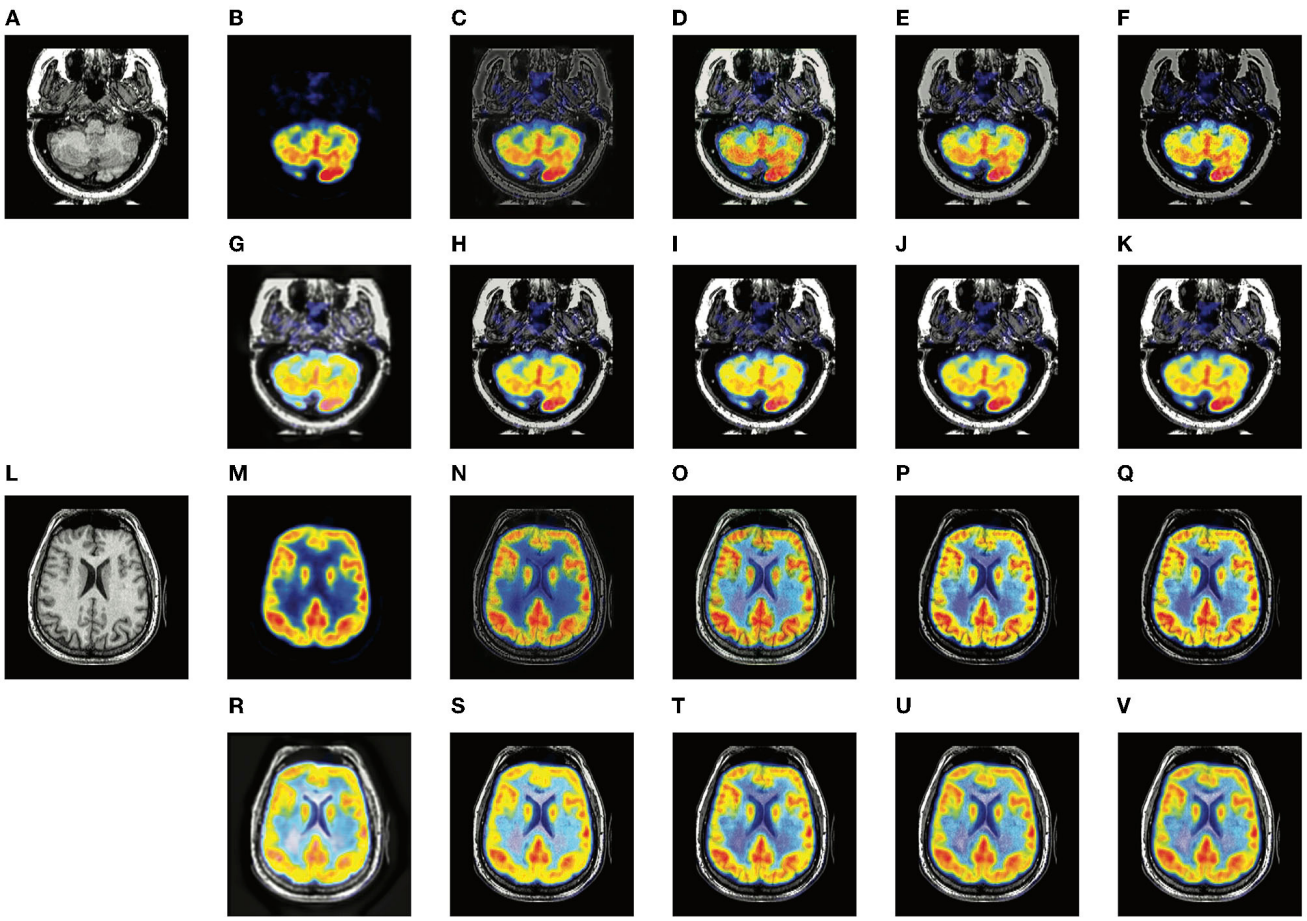
Experiments results of proposed method with six deep learning-based methods on two typical MRI and PET image pairs. **(A,L)** MRI images; **(B,M)** PET images; **(C,N)** fused results of DDcGAN; **(D,O)** fused results of EMFusion; **(E,P)** fused results of IFSR; **(F,Q)** fused results of U2Fusion; **(G,R)** fused results of DRF; **(H,S)** fused results of SEDR; **(I,T)** fused results of proposed-add; **(J,U)** fused results of proposed-max; **(K,V)** fused results of proposed-l1.

Two typical pairs of MRI-CT images and two typical pairs of MRI-PET images are presented in Figures 4, 5, respectively. MRI images depict accurate and abundant soft tissue, CT images provide dense structures with less distortion, and PET images provide a detailed function of focus of infection and metabolism information. From the visual results, it can be seen that the fused images of DDcGAN show a lot of distorted information in Figures 4C,N, and it almost loses all the MRI information in Figures 5C,N. This is caused by the instability of GAN, and it is inappropriate for the adopted loss function to represent the information of MRI as gradients only. The fused images of IFSR, U2Fusion, and SEDR lose much saliency of soft tissue and dense structures and present a low contrast on the whole. DRF provides relatively blurred results and loses a lot of sharp details. Besides, the color of the PET image is severely distorted in its fused results. Among these methods, U2Fusion measures the amount of gradient in each source image to assign the weights of the loss function, realizing the adaptive control of similarity between fused images and source images. However, such assignments are conducted evenly on the whole image, thus leading to the degradation of image contrast. SEDR maps both source images into the same space, ignoring the unique modality information. Fusion operations on such features can lead to loss of significance. IFSR and DRF all take into account the disentanglement, however, they lose the consideration of the corresponding relationship of source images in different positions. Moreover, DRF compresses the modality information into a vector, which can cause the distortion of spatial information. On the whole, EMFusion and the proposed method taking different fusion strategies can all provide the fused image with abundant details and clear edges. EMFusion is able to enhance the PET information with MRI details, while the proposed method can show the CT and MRI information with higher brightness.

### 4.5. Quantitative Evaluation

The quantitative results of MRI-CT and MRI-PET fusion tasks are presented in Tables 4, 5. From the average values, DDcGAN obtains the best *SD* and *SF* in the MRI-CT task, which means the fused results a higher dispersion degree of the gray value and many details of high frequency. However, SD and *SF* can only reflect the quality of the fused image itself and fails to measure the information transferred from source images, meanwhile, Figures 4C,N contains much distorted information. SEDR is able to achieve the best *Q*_*MI*_ and *Q*_*NCIE*_ in the MRI-CT fusion task, which reveals the fused image shows a higher correlation with both source images. But it shows weaker performance on MRI-CT tasks, as Figures 4H,S shows that the image contrast is degraded. EMFusion shows the best performance on *Q*_*G*_, *Q*_*S*_, and *Q*_*CB*_ in the MRI-PET task. Compared to other methods, EMFusion makes use of the MRI images to enhance the details of chrominance channels in PET images, instead of only fusing the luminance channel separately from the chrominance channels. Thus, EMFusion is capable of presenting high-quality color information with clear gradients. The proposed method which adopts different fusion strategies is able to achieve the best results on *Q*_*M*_, *MSSSIM*, *SCD*, and *VIFF* in both tasks, and it also shows second-best performance on the most of the rest metrics. By comparing the three different fusion strategies on complementary features quantitatively and qualitatively, the addition strategy is good at showing more texture details as it directly combines all the information together. Thereby, it can also maintain the integral structure in both MRI-CT and MRI-PET tasks. The L1-selection strategy shows better performance in MRI-CT as it can adaptively assign the fusion weights. Max-selection can preserve the position with strong pixel intensity, however, it cannot avoid the loss of information to some degree. From the variance, the proposed method shows larger fluctuation than other methods on SD and SF. We assume this is because the content of training images in different folds of dataset can affect the generalization ability of a neural network to some degree. Besides, SD and SF evaluate the image quality by measuring the statistical features of the fused image, without considering the source image. To make a comprehensive assessment, the two metrics should be combined with the rest metrics which reflect the transfer ability of the fusion methods. In general, the proposed method presents a good ability in transferring edge details and preserving structural information, able to provide images with good visual quality. Such advantage is attributed to the disentanglement of redundant and complementary features, which makes the fusion process more accurate.

**Table 4:** The quantitative evaluation of different comparison methods on the MRI-CT dataset.

**Methods**	**Objective metrics**										
	***SD***	***SF***	***Q*_*MI*_**	***Q*_*NCIE*_**	***Q*_*G*_**	***Q*_*M*_**	***Q*_*S*_**	***MSSSIM***	***SCD***	***Q*_*CB*_**	***VIFF***
DDcGAN	88.13 ± 0.89	32.40 ± 0.57	0.58 ± 0.01	0.80 ± 0.00	0.57 ± 0.01	0.17 ± 0.00	0.25 ± 0.01	0.71 ± 0.00	1.24 ± 0.02	0.23 ± 0.01	0.25 ± 0.00
EMFusion	80.36 ± 0.59	20.76 ± 0.30	0.81 ± 0.01	0.81 ± 0.00	0.72 ± 0.01	0.16 ± 0.00	0.81 ± 0.00	0.89 ± 0.00	1.20 ± 0.05	0.67 ± 0.02	0.42 ± 0.01
IFSR	68.91 ± 0.46	19.81 ± 0.42	0.67 ± 0.01	0.81 ± 0.01	0.54 ± 0.01	0.11 ± 0.01	0.62 ± 0.00	0.89 ± 0.01	1.01 ± 0.03	0.34 ± 0.00	0.40 ± 0.00
U2Fusion	58.77 ± 0.38	21.06 ± 0.29	0.68 ± 0.01	0.81 ± 0.00	0.67 ± 0.01	0.13 ± 0.00	0.34 ± 0.00	0.89 ± 0.01	0.76 ± 0.03	0.28 ± 0.00	0.35 ± 0.01
DRF	75.42 ± 0.70	9.58 ± 0.14	0.51 ± 0.01	0.80 ± 0.00	0.22 ± 0.01	0.11 ± 0.00	0.22 ± 0.00	0.74 ± 0.00	1.19 ± 0.08	0.18 ± 0.00	0.31 ± 0.01
SEDR	63.78 ± 0.68	20.75 ± 0.35	0.81 ± 0.01	0.81 ± 0.00	0.56 ± 0.02	0.14 ± 0.00	0.32 ± 0.01	0.87 ± 0.00	0.85 ± 0.05	0.24 ± 0.00	0.36 ± 0.01
Proposed-add	84.92 ± 2.64	27.23 ± 0.27	0.80 ± 0.01	0.81 ± 0.00	0.72 ± 0.01	0.16 ± 0.02	0.82 ± 0.01	0.91 ± 0.01	1.37 ± 0.10	0.66 ± 0.01	0.45 ± 0.03
Proposed-max	80.48 ± 2.62	30.23 ± 1.05	0.80 ± 0.01	0.81 ± 0.00	0.74 ± 0.01	0.21 ± 0.03	0.82 ± 0.01	0.88 ± 0.01	1.16 ± 0.07	0.68 ± 0.01	0.41 ± 0.04
Proposed-l1	81.59 ± 2.49	29.28 ± 1.43	0.81 ± 0.02	0.81 ± 0.00	0.75 ± 0.01	0.22 ± 0.01	0.82 ± 0.01	0.88 ± 0.01	1.20 ± 0.10	0.68 ± 0.00	0.41 ± 0.04

*The evaluation is shown as average±variance of testing results on 10-group image pairs, which the proposed method adopts for 10-fold verification experiments*.

**Table 5:** The quantitative evaluation of different comparison methods on the MRI-PET dataset.

**Methods**	**Objective metrics**										
	***SD***	***SF***	***Q*_*MI*_**	***Q*_*NCIE*_**	***Q*_*G*_**	***Q*_*M*_**	***Q*_*S*_**	***MSSSIM***	***SCD***	***Q*_*CB*_**	***VIFF***
DDcGAN	57.93 ± 0.23	22.93 ± 0.14	0.53 ± 0.00	0.81 ± 0.00	0.53 ± 0.01	0.16 ± 0.01	0.57 ± 0.00	0.80 ± 0.00	0.67 ± 0.00	0.34 ± 0.00	0.35 ± 0.00
EMFusion	75.97 ± 0.11	32.03 ± 0.06	0.68 ± 0.00	0.81 ± 0.00	0.77 ± 0.00	0.42 ± 0.02	0.91 ± 0.00	0.91 ± 0.00	1.02 ± 0.01	0.62 ± 0.00	0.46 ± 0.00
IFSR	69.21 ± 0.15	25.29 ± 0.09	0.60 ± 0.00	0.81 ± 0.00	0.63 ± 0.00	0.15 ± 0.01	0.80 ± 0.00	0.92 ± 0.00	1.17 ± 0.02	0.55 ± 0.00	0.51 ± 0.00
U2Fusion	72.90 ± 0.07	26.05 ± 0.05	0.64 ± 0.00	0.80 ± 0.00	0.63 ± 0.00	0.17 ± 0.00	0.70 ± 0.01	0.89 ± 0.00	1.29 ± 0.00	0.60 ± 0.01	0.51 ± 0.00
DRF	71.40 ± 0.65	12.76 ± 0.09	0.46 ± 0.00	0.80 ± 0.01	0.34 ± 0.00	0.10 ± 0.00	0.46 ± 0.00	0.74 ± 0.00	0.82 ± 0.02	0.38 ± 0.00	0.36 ± 0.00
SEDR	84.47 ± 0.10	31.01 ± 0.06	0.76 ± 0.00	0.81 ± 0.00	0.73 ± 0.00	0.34 ± 0.01	0.84 ± 0.00	0.92 ± 0.00	1.52 ± 0.02	0.57 ± 0.00	0.55 ± 0.00
Proposed-add	89.28 ± 1.21	34.41 ± 0.27	0.76 ± 0.01	0.81 ± 0.00	0.77 ± 0.00	0.51 ± 0.08	0.80 ± 0.01	0.94 ± 0.00	1.65 ± 0.03	0.50 ± 0.01	0.59 ± 0.00
Proposed-max	86.84 ± 1.16	35.24 ± 0.49	0.72 ± 0.12	0.81 ± 0.00	0.70 ± 0.11	0.39 ± 0.20	0.77 ± 0.13	0.87 ± 0.08	1.31 ± 0.21	0.54 ± 0.02	0.49 ± 0.07
Proposed-l1	88.72 ± 2.17	36.05 ± 1.43	0.72 ± 0.15	0.81 ± 0.00	0.70 ± 0.14	0.38 ± 0.09	0.74 ± 0.06	0.86 ± 0.07	1.36 ± 0.17	0.52 ± 0.04	0.49 ± 0.06

*The evaluation is shown as average±variance of testing results on 10-group image pairs, which the proposed method adopts for 10-fold verification experiments*.

### 4.6. Time Cost Comparison

The running efficiency of a method is an important index to measure the performance as well. The average running time of different methods on all the test MRI-CT and MRI-PET image pairs is presented in Table 6. All methods are conducted on the same platform with Intel Core i7-6850K CPU and GeForce GTX 1080Ti GPU. From the time cost comparison, the proposed method is the most efficient than other comparison methods.

**Table 6:** Time cost comparison.

**Methods**	**DDcGAN**	**EMFusion**	**IFSR**	**U2Fusion**	**DRF**	**SEDR**	**Proposed**
Image size	256 × 256	256 × 256	256 × 256	256 × 256	256 × 256	256 × 256	256 × 256
Time cost	0.589s	0.448s	2.499s	0.086s	1.176s	0.900s	0.037s

## 5. Conclusion

In this article, a disentangled representation based brain image fusion method is proposed. A three-branch auto-encoder architecture is designed to fully explore the significant features and correlations benefit of image fusion tasks, dealing with the unique modality characteristics. Based on the prior knowledge of complementary and redundant relationships, a complementary group lasso penalty is proposed for effective disentangled representation learning, which is able to separate the discriminative modality information from the structure information. The disentangled representations show better interpretability to allow simple fusion strategies and improve the precision of fusion results. The experiments on MRI-CT and MRI-PET fusion tasks demonstrate the effectiveness of the proposed method in retaining structure and details, as well as presenting good visual quality.

Nevertheless, the proposed method only focuses on the fusion of gray-scale images, and the chrominance channels of PET images are kept and directly combined with the fused gray-scale images, which leads to the degradation of texture information. In the future, how to embed the chrominance channels into a disentangled framework should be considered. Code and pre-trained models are available at https://github.com/qqchong/A-Disentangled-Representation-based-Brain-Image-Fusion-via-Group-Lasso-Penalty.

## Data Availability Statement

Publicly available datasets were analyzed in this study. This data can be found at: http://www.med.harvard.edu/aanlib/. Code and pre-trained models are available at https://github.com/qqchong/A-Disentangled-Representationbased-Brain-Image-Fusion-via-Group-Lasso-Penalty.

## Author Contributions

AW and ZZ conceived the study. AW and XL designed the specific method. ZZ, XL, and X-JW analyzed the experiment data. AW wrote the draft. All authors gave critical revision and consent for this submission.

## Funding

This study was supported in part by the National Natural Science Foundation of China under Grant No. 61772237, and the Six Talent Peaks Project in Jiangsu Province under Grant XYDXX-030.

## Conflict of Interest

The authors declare that the research was conducted in the absence of any commercial or financial relationships that could be construed as a potential conflict of interest.

## Publisher's Note

All claims expressed in this article are solely those of the authors and do not necessarily represent those of their affiliated organizations, or those of the publisher, the editors and the reviewers. Any product that may be evaluated in this article, or claim that may be made by its manufacturer, is not guaranteed or endorsed by the publisher.
